# Case Report: Recanalization of Branch Retinal Artery Occlusion Due to Microthrombi Following the First Dose of SARS-CoV-2 mRNA Vaccination

**DOI:** 10.3389/fphar.2022.845615

**Published:** 2022-03-24

**Authors:** Min Seung Kang, Sang Yoon Kim, Han Jo Kwon

**Affiliations:** ^1^ Research Institute for Convergence of Biomedical Science and Technology, Pusan National University Yangsan Hospital, Yangsan, South Korea; ^2^ Department of Ophthalmology, Pusan National University Yangsan Hospital, Yangsan, South Korea; ^3^ Biomedical Research Institute, Pusan National University Hospital, Busan, South Korea

**Keywords:** BNT162b2, branch retinal artery occlusion, case report, COVID-19, mRNA vaccine, SARS-CoV-2, spontaneous recanalization, thrombus

## Abstract

**Background:** We report on a patient with a branch retinal artery occlusion (RAO) and its recanalization based on multimodal retinal and angiographic images after he was administered the first dose of the SARS-CoV-2 mRNA vaccine.

**Case summary:** A 64-year-old man complained of a right, painless, inferior field defect 3 days after the first dose of BNT162b2 vaccination. Fundus examination revealed decolorization of the right upper macula, including microthrombi in the superior proximal branch of the retinal artery. Optical coherence tomography angiography revealed upper macular hypoperfusion. Fluorescein angiography revealed prolonged arteriovenous transit to the macula. After paracentesis with antiplatelet medications, the artery was recanalized as the thrombi dissolved, and the right visual field was recovered. Re-occlusion did not occur during the 3 months after the second mRNA vaccination.

**Conclusion:** Non-embolic thrombotic RAO may develop shortly after the SARS-CoV-2 mRNA vaccine. Ophthalmologists should consider RAO as a possible post-vaccination adverse event. The temporal association between mRNA vaccination and RAO onset with evidence of microthrombi might provide additional clues to elucidate the unpredictive arterial thrombosis following SARS-CoV-2 mRNA vaccination.

## Introduction

The development and distribution of the coronavirus disease (COVID-19) vaccination program is unique compared to prior pandemic eras. By December 2021, over 8.5 billion doses of the vaccines against severe acute respiratory syndrome coronavirus-2 (SARS-CoV-2) had been administered to the world population (Mathieu et al., 2021). Thromboembolic adverse events (AEs) after COVID-19 or SARS-CoV-2 immunization have been discussed worldwide (Avila et al., 2021; Brazete et al., 2021). Characteristically, thrombosis and thrombocytopenia syndrome after adenoviral-vectored COVID-19 vaccination, also known as vaccine-induced immune thrombotic thrombocytopenia, indicates a high mortality rate, is predominant in women under 45 years of age, and manifests within 2 weeks of vaccination without prothrombotic risk factors (Palaiodimou et al., 2021). In addition, a case of thrombosis with thrombocytopenia syndrome following the SARS-CoV-2 mRNA vaccination has now been announced (Sangli et al., 2021). Due to this finding, ophthalmologists remain alert for possible ocular AEs after SARS-CoV-2 vaccination. Several vision-threatening ocular AEs have been reported, including central retinal vein occlusion (RVO), acute macular neuroretinopathy, and panuveitis (Bialasiewicz et al., 2021; Endo et al., 2021; Ng et al., 2021). In contrast to the reversible course of ocular AEs caused by retinal venocapillary occlusion or inflammatory vasculitis, retinal arterial occlusion can lead to more devastating outcomes due to ischemic retinal damage and subsequent permanent vision loss. We report the first case of recanalization of branch retinal artery occlusion (RAO) following the first dose of mRNA vaccination with detailed multimodal ophthalmic images.

## Case Description

On April 28, 2021, a 64-year-old Korean man was referred for a retinal specialist review with complaints of right sudden visual deterioration with an inferior altitudinal field defect 3 days after receiving the first dose of the BNT162b2 vaccine (Lot Number: ET9096, Tozinameran, Pfizer-BioNTech) on April 21. He had no history of COVID-19, and the real-time reverse transcriptase polymerase chain reaction test was also negative at the initial visit. Initial vital signs were heart rate, 89/minute (min); blood pressure, 130/80 mmHg; respiratory rate, 26 breaths/min; oxygen saturation, 99%; electrocardiogram, normal sinus rhythm. Five years ago, he was diagnosed with type 2 diabetes and hypertension and had been treated with metformin hydrochloride 750 mg once per day (q.d.), glimepiride 1 mg q.d., gemigliptin tartrate sesquihydrate 50 mg q.d., and losartan/hydrochlorothiazide 50/12.5 mg q.d. at a local internal medicine clinic. He had no chest pain, cardiac arrhythmia, coronary heart disease, or myocardial or cerebral infarction history.

### Initial Ophthalmic Examination

**FIGURE 1:**
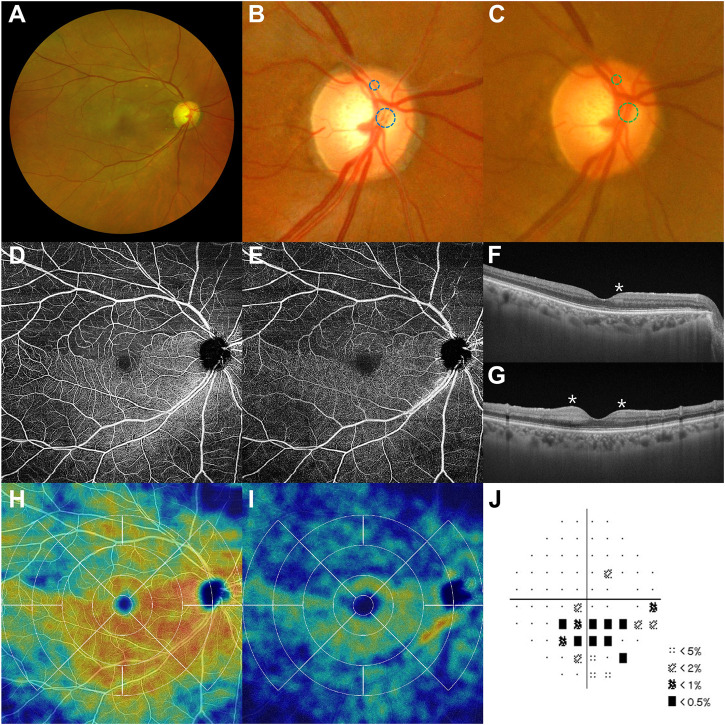
Branch retinal artery occlusion after the first dose of the SARS-CoV-2 mRNA vaccine. Multimodal imaging findings at initial visit including fundus photographs, optical coherence tomography (OCT, DRI OCT-1 Atlantis; Topcon Corp., Tokyo, Japan), and OCT angiography (Cirrus HD-OCT model 5,000 with AngioPlex™; Carl Zeiss Meditec, Inc., Dublin., CA, United States) on day 7 post-vaccination. **(A,B)** A right upper section of the macula is pale. Multiple thrombi (blue dashed circles) are found in the superior branches of the right central retinal artery. **(C)** These thrombi were absent in the arteries (green dashed circles) 6 years ago. **(D,E)** SCP and DCP on the upper hemisphere are barely visible because of attenuated decorrelation signals. **(F,G)** Inner retinal edema (asterisks), a characteristic finding of retinal artery occlusion, exists near the fovea. **(H,I)** Superior macular perfusion to SCP and DCP decreases rather than the inferior side. **(J)** Automated Humphrey perimetry reveals an arcuate anopsia to the inferior field. DCP, deep capillary plexus; SCP, superficial capillary plexus.

The initial best-corrected visual acuity (BCVA) was 20/50 in the right eye (oculus dexter) and 20/20 in the left (oculus sinister). The intraocular pressures (IOP) were 18/17 mmHg with normal pupillary responses. Anterior chamber cells and other media opacities were not detected. On day 7 post-vaccination, a fundus examination revealed whitening of the upper hemispheric macula and multiple microthrombi in the superior branch of the central retinal artery (Figures 1A, B). The patient underwent successful cataract surgery 6 years ago, and a healthy retinal vasculature originating from the optic disc was evident on preoperative fundus photography (Figure 1C). Superficial and deep capillary plexus (SCP and DCP, respectively) examined with optical coherence tomography (OCT) angiography (Cirrus HD-OCT model 5,000 with AngioPlex™; Carl Zeiss Meditec, Inc., Dublin, CA, United States) showed a lack of decorrelation signals in the upper macula (Figures 1D, E [SCP], [DCP]). However, the choriocapillaris and choroid signals were intact. Cross-sectional OCT (OCT, DRI OCT-1 Atlantis; Topcon Corp., Tokyo, Japan) revealed inner retinal edema dominantly at the parafovea (Figures 1F, G). The upper macular perfusion on the SCP and DCP were decreased in the OCT angiography perfusion map using the Macular Density algorithm (v0.7.3, ARI Network Hub, https://arinetworkhub.com/) (Figures 1H, I [SCP], [DCP]). Visual field defects corresponding to reduced perfusion areas were noticeable in the pattern deviation map on the Humphrey Field Analyzer (Carl Zeiss Meditec, Inc., Dublin, CA, United States) (Figure 1J).

**FIGURE 2:**
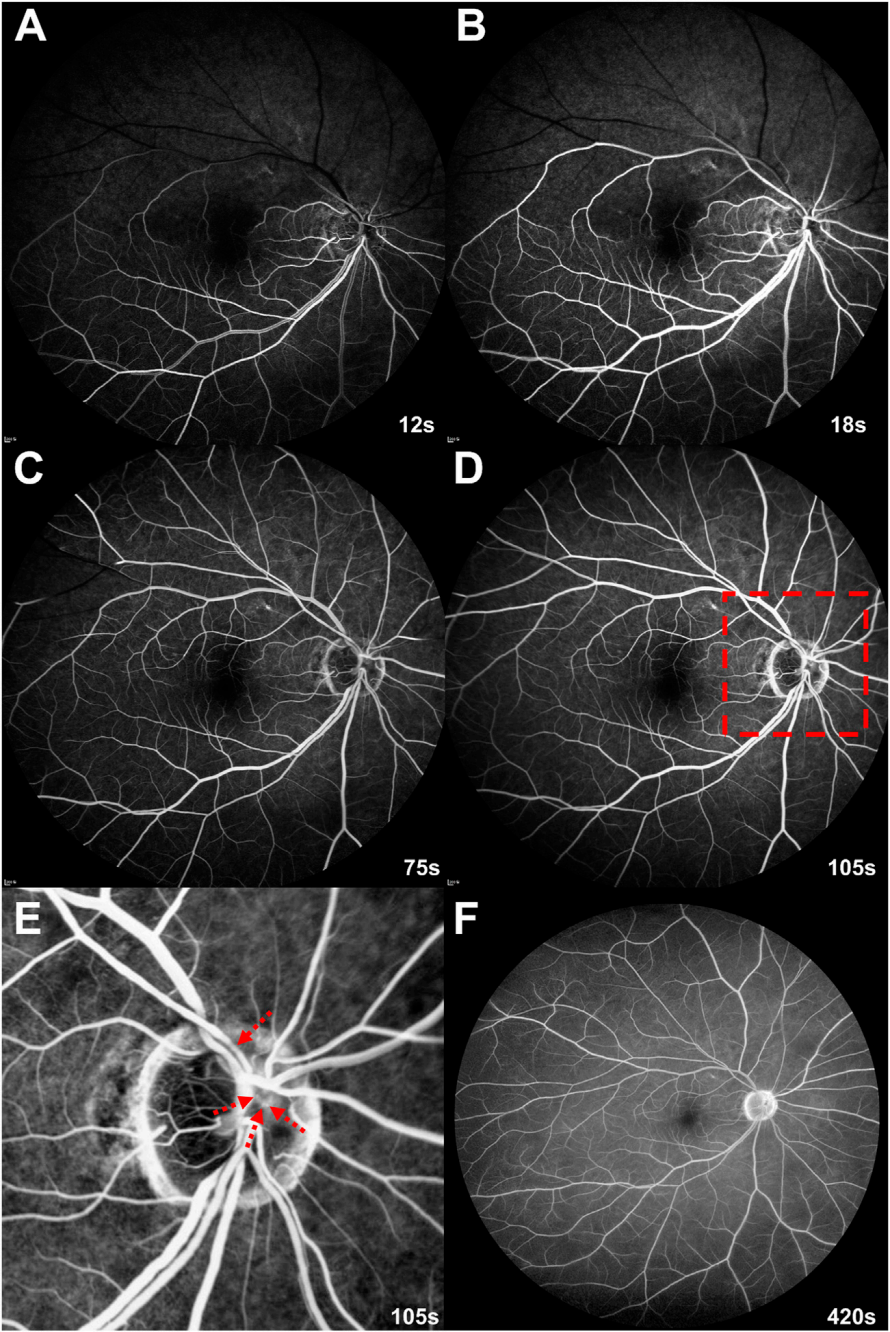
Initial fluorescein angiography on day 7 post-vaccination **(A–D)** Arterial filling delay to the upper branches protracts venous return. **(E)** An enlarged image of the red dashed rectangle on **(D)**. Fluorescein angiography at the venous phase discriminates fluorescein-stained microthrombi (red dashed arrows) in the upper proximal branches of the right retinal arteries. **(F)** Until the late phase, dye leakage and retinal vasculitis are absent.

Fluorescein angiography showed a filling delay in the superotemporal and superonasal arterial branches, but typical background fluorescence from the choroid (Figure 2A). The arteriovenous transit time of the inferior branch was 8 s, while that of the superior branch was delayed to 65 s (Figures 2B, C). Laminar flow in the superior retinal vein disappeared 95 s after the first appearance of the dye in the retinal artery (Figure 2D). At that time, four microthrombi were detected in the inner cavities of the superotemporal and superonasal proximal retinal arteries (Figure 2E). Until the late phase of the fluorescein angiography, retinal vascular leakage was absent (Figure 2F). Ultimately, he was diagnosed with branch RAO (BRAO) due to microthrombi following SARS-CoV-2 mRNA vaccination and referred to a stroke center to investigate another lesion or source of microthrombi.

### Neurological Evaluation, Brain Imaging, and Treatments

**FIGURE 3:**
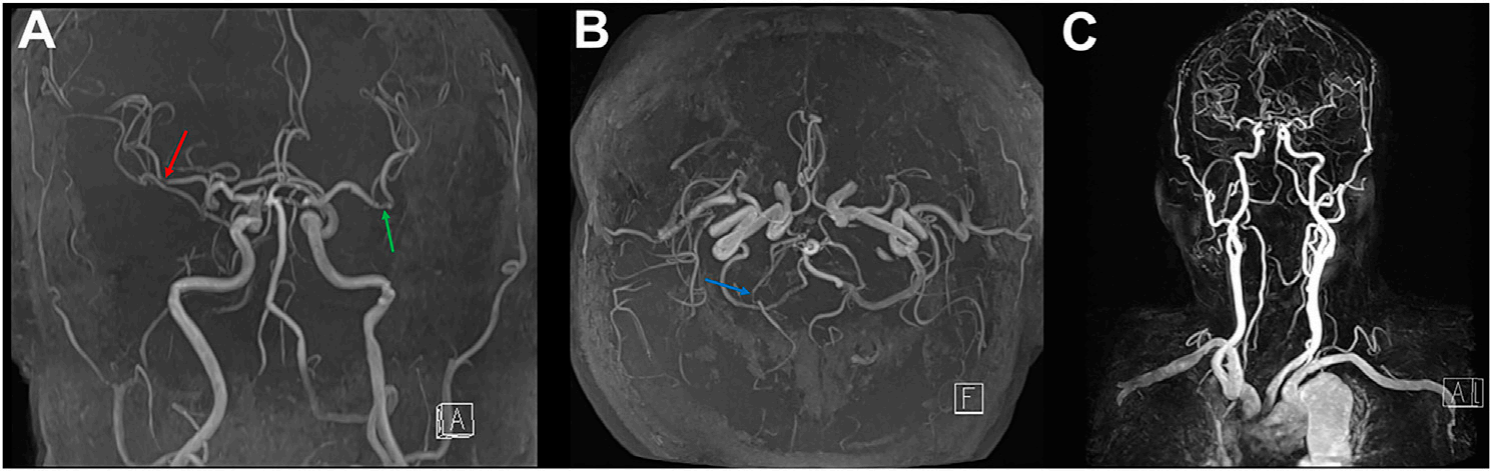
Contrast-enhanced magnetic resonance angiography for the brain and carotid on day 7 post-vaccination **(A,B)** Brain magnetic resonance angiography shows focal stenosis at the right (red arrow) and left (green arrow) insular segment (M2) on middle cerebral arteries and post-communicating segment (P2, blue arrow) on the right posterior cerebral artery. **(C)** No vascular abnormality detects on carotid magnetic resonance angiography.

Neurologists examined him for a possible ischemic stroke, and no specific findings were observed except for right blurry vision. Contrast-enhanced magnetic resonance angiography (MRA) of the brain and carotid arteries revealed focal stenosis of the bilateral M2 and right P2 branches (Figures 3A, B). No stenosis was noted on carotid MRA (Figure 3C), and diffusion-weighted imaging and fluid-attenuated inversion recovery on MRI were unremarkable. On the subsequent evaluation of coagulation markers, the creatine kinase MB isoenzyme (CK-MB) was elevated, but the high-sensitivity troponin I (hsTnI) assay was within the reference range (Supplementary Table S1). On an automated hematology analyzer, the platelet count was 215,000/μL, and mean platelet volume and platelet distribution width were 9.5 fL and 10.0%, respectively. Echocardiography revealed no specific findings. The neurologist added aspirin 100 mg and atorvastatin calcium trihydrate 20 mg q.d. to the patient’s medication. An ophthalmologist (HJK) performed anterior chamber paracentesis to increase ocular perfusion pressure and administered brimonidine tartrate (1.5 mg) and dorzolamide/timolol (20/5 mg/ml) twice per day (b.i.d.).

### Assessment of Multimodal Imaging After Treatment

Two days after the initial treatment (April 30, day 9 post-vaccination), the right BCVA was 20/30, and the right IOP dropped to 8 mmHg. Although some areas of field defect remained, his visual discomfort slightly improved as thrombi progressively dissolved within the arterial cavities (Supplementary Figure S1). Nine days after the initial treatment (May 7, day 16 post-vaccination), the size of the remnant thrombi continued to decrease. The right BCVA was 20/25, and the IOP was maintained at 8 mmHg. After explaining the possibility of re-occlusion or cerebral infarction, the patient was administered a second dose of the same vaccine (Lot Number: EX6564) on May 12 (day 21 post-vaccination).

**FIGURE 4:**
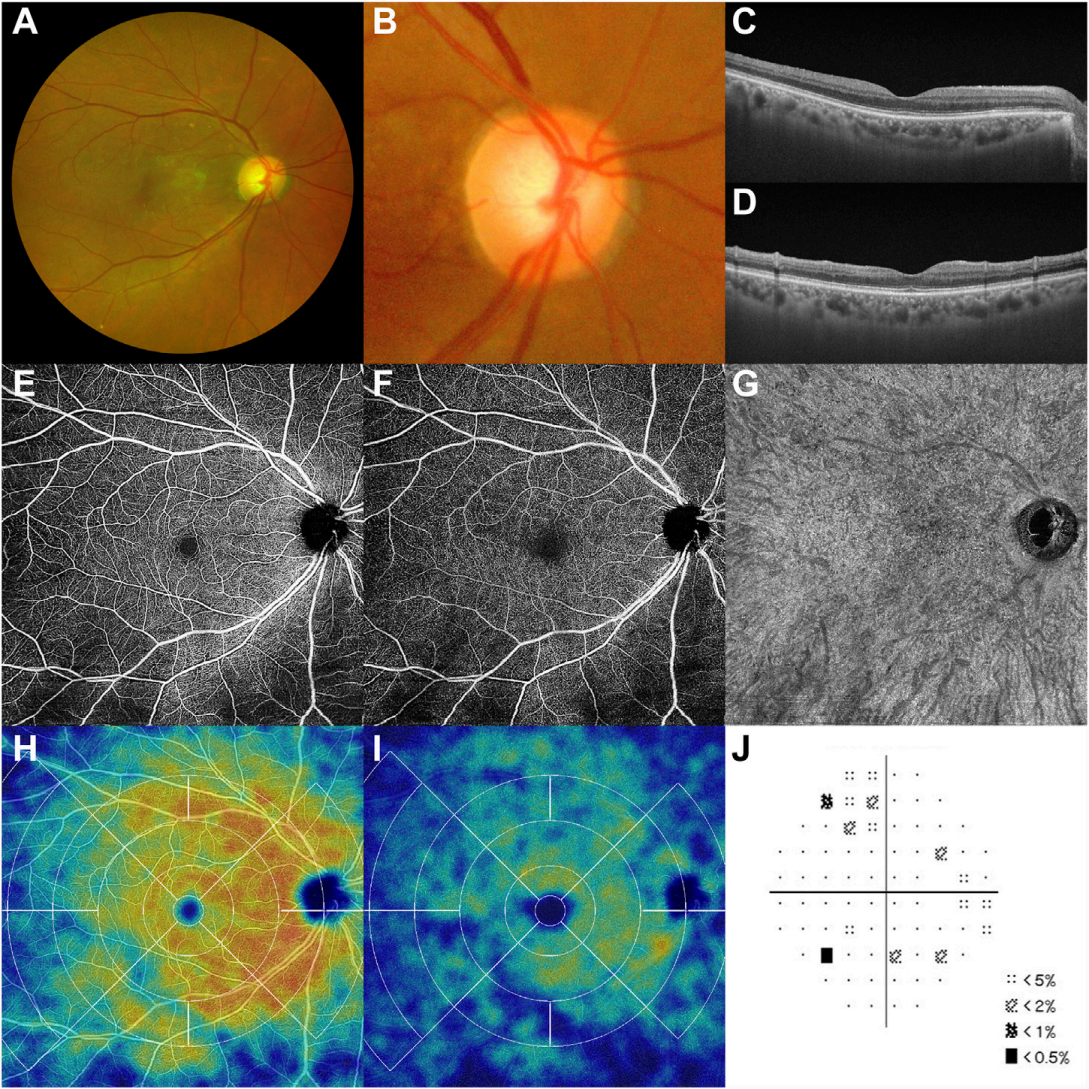
Follow-up multimodal images after treatments and the second dose of the SARS-CoV-2 mRNA vaccine **(A,B)** With recanalization and degraded microthrombi, macular ischemia disappears, and the color of the upper macula turns to normal on day 30 post-first vaccination. **(C–G)** Decorrelation signals from SCP and DCP revive with mitigated inner retinal edema, and choroidal vasculature is intact. **(H,I)** Upper macular perfusion increases in both SCP and DCP. **(J)** Along with the anatomical improvements, inferior visual fields recover partially. DCP, deep capillary plexus; SCP, superficial capillary plexus.

Twenty-three days after the initial treatment (May 21, 30 days post-vaccination), the right BCVA increased to 20/20. All follow-up multimodal images indicated reperfusion from the RAO. Retinal whitening and microthrombi were diminished (Figure 4A), and narrowed diameter of the proximal portion of the superior retinal arterial branch (Figure 4B). The inner retinal edema also disappeared (Figures 4C, D). Decreased decorrelation signals of the SCP and DCP were also restored on OCT angiography (Figures 4E, F [SCP], [DCP]), and those of the choriocapillaris remained intact (Figure 4G). On the OCT angiography perfusion map, the previous hypoperfusion of the SCP and DCP showed spontaneous reperfusion (Figures 4H, I [SCP], [DCP]). The inferior field deficits were concurrently resolved (Figure 4J). This reperfusion and 20/20 right visual acuity were maintained until August 30 (day 131 post-vaccination). Significant visual symptoms, clinical courses, and important medical events are summarized in a timeline format (Supplementary Figure S2).

## Discussion

Vaccine-induced immune thrombotic thrombocytopenia with severe multiorgan involvement is a representative systemic AE that can develop 4–29 days after a viral-vector-type SARS-CoV-2 vaccine and is presumed to be associated with autoimmunity against platelet factor 4 (Brazete et al., 2021; Greinacher et al., 2021; Schultz et al., 2021; Scully et al., 2021). In cases of the SARS-CoV-2 mRNA vaccine, thrombotic AEs, such as cerebral venous sinus thrombosis, deep vein thrombosis, and pulmonary embolism, have also been reported (Brazete et al., 2021; European Medicines Agency, 2021). In particular, myocardial microthrombi with acute chest pain occurred 19 days after SARS-CoV-2 mRNA vaccination without elevation of D-dimer and haptoglobin levels (Aikawa et al., 2021). They described that erythrocyte-rich microthrombi occluded capillary vessels and were accompanied by extravasation of erythrocytes without inflammatory cell infiltration through endomyocardial biopsy, which is analogous to our case, except for the invasion of other organs.

RAO, a serious thromboembolic event, is a type of central nervous system infarction and a concomitant ophthalmic emergency that occurs in the end artery supplying blood flow to the superficial retina (Sacco et al., 2013). BRAO, where the temporal branches of the central retinal artery are predominantly occluded, accounts for approximately 40% of all RAOs (Ros et al., 1989). The causes of BRAO can be subdivided into embolic sources and, less frequently, non-embolic causes. Non-embolic BRAO is associated with inflammation, infection, vasospasm, and hypercoagulation (Ros et al., 1989). Unfortunately, no confirmative method prevents RAO and induces early reperfusion for visual recovery.

Retinal thrombotic AEs have been reported as predominantly RVO with a short time window following SARS-CoV-2 vaccination. Its clinical features are reversible, except in ischemic central RVO (Bialasiewicz et al., 2021; Endo et al., 2021). The vascular density of the peri-papillary region was significantly decreased on OCT angiography in patients with COVID-19 and associated with clinical severity markers (Hohberger et al., 2021). If thrombosis develops in the central retinal artery, ophthalmologists, and vaccinated individuals should remain vigilant because of irreversible visual deterioration, particularly since the incidence of spontaneous recanalization from occlusion is as low as approximately 15% (Ahn et al., 2015). Three cases of RAO that arose 2–12 days after SARS-CoV-2 mRNA vaccination have been reported recently. However, the changes and outcomes of visual acuity and field were not discussed (Girbardt et al., 2021; Ikegami et al., 2021). In one case, BRAO occurred in a 39-year-old man 4 days after the second dose of the BNT162b2 vaccine without thrombocytopenia (Girbardt et al., 2021). Two additional cases combined with RAO and RVO were presented after the second dose of the mRNA-1273 and BNT162b2 vaccines (Girbardt et al., 2021; Ikegami et al., 2021). The common features of these RAO cases, along with this case, were non-embolic RAO, not accompanied by carotid plaque or stenosis, mRNA of SARS-CoV-2 vaccine, and rapid development within 2 weeks after vaccination. Based on this BRAO and other RAO cases following SARS-CoV-2 vaccination, we cannot exclude the SARS-CoV-2 mRNA vaccine as a cause for *de novo* thrombosis. However, the exact mechanism has not been elucidated. Autoimmunity for the S-protein may trigger thrombosis within a few days after the mRNA vaccine.

Interestingly, several cases of RAO have been reported after COVID-19. Montesel et al. (2020) reported the first case of central RAO (CRAO) with elevated inflammatory and procoagulant markers persisting 2.5 months after COVID-19. In addition to cases of CRAO, BRAO, and ophthalmic artery occlusion following COVID-19 (Acharya et al., 2020; Dumitrascu et al., 2020; Uzun et al., 2021), there have been reports of combined presentations of CRAO and ischemic stroke (Been Sayeed et al., 2021). Between 1 week and 5 months of hospitalization for COVID-19, RAO can present late thrombotic events as a complication of a severe inflammatory response with elevated D-dimer levels, increased C-reactive protein, and thrombocytopenia. The clinical patterns of SARS-CoV-2 vaccine-associated RAO are significantly different from RAO developing after COVID-19. The time interval from vaccination to RAO onset is relatively short without marked abnormalities of laboratory tests, such as increased D-dimer level and C-reactive protein.

Although this report is a single anecdote with a short observation period, noninvasive multimodal retinal images vividly demonstrate the emergence or disappearance of microthrombi and the associated decrease or improvement of flow to the retina. Retinal multimodal imaging techniques can swiftly identify unpredictable ocular AEs following COVID-19 and vaccination. Impairment of retinal microcirculation may reflect problems in the systemic vessel system, and microthrombi might also be involved in focal stenosis on brain MRA in this patient. If circulating microthrombi reach the retinal and cerebral arteries, the consequences of arterial obstruction can lead to retinal or brain tissue hypoxia. Therefore, we cannot simply ignore these thrombotic AEs from the perspective of “Time is Tissue” (Varma et al., 2013).

Considering the date of his first visit before the concept of thrombosis with thrombocytopenia syndrome was established (Brighton Collaboration, 2021), this retinal thrombotic AE did not lead to evaluate serum level of D-dimer and detect anti-platelet factor 4 antibodies. There was no evidence of atrial fibrillation in his previous symptoms, electrocardiogram, or echocardiography. Nonetheless, Holter monitoring can considerably increase the detection rate of atrial fibrillation (Callizo et al., 2017), one of the causes of RAO; thus, not performing heart rhythm monitoring is another limitation in this report.

In conjunction with the well-known noninvasive therapies for RAO, acetylsalicylic acid and lipid-lowering medications can be used to inhibit additional platelet deposition and induce recanalization from arterial occlusion due to microthrombi. This case should not be regarded as a reason to avoid SARS-CoV-2 vaccination but rather a caution of possible AE within a specific temporal window.

## Data Availability

The original contributions presented in the study are included in the article/Supplementary Material, further inquiries can be directed to the corresponding author.
