# Supplementary material for: Case Report: Recanalization of Branch Retinal Artery Occlusion Due to Microthrombi Following the First Dose of SARS-CoV-2 mRNA Vaccination
Source: Front Pharmacol. 2022 Mar 24;13:845615. doi: 10.3389/fphar.2022.845615 (PMC8988066; doi:10.3389/fphar.2022.845615)
Supplement: Supplementary file 1 [file Table1.DOCX]

**SUPPLEMENTAL TABLE 1. Laboratory test results with coagulation markers at initial visit**

| **Markers** | **Results** | **Reference ranges** |
| --- | --- | --- |
| PT | 12.9 | 11.5–14.5 sec |
| PT INR | 0.96 | 0.80–1.20 |
| aPTT | 32.7 | 28.3–43.8 sec |
| hsTnI | 7.53 | 0–19.8 pg/mL |
| CK-MB | 9.3 | 0.5–3.1 ng/mL |
| BNP | 84 | 0–100 pg/μL |
| WBC | 10.16 | 4.0–11.0 × 10^3^/μL |
| Hemoglobin | 16.2 | 13.5–17.5 g/dL |
| Platelet | 215 | 140–400 × 10^3^/μL |
| MPV | 9.5 | 9.2–12.0 fL |
| PDW | 10.0 | 9.6–15.2 % |
| hsCRP | 0.09 | 0–0.5 mg/dL |

aPTT, activated partial thromboplastin time; BNP, B-type natriuretic peptide; CK-MB, creatine kinase-MB; INR, international normalized ratio; hs-CRP, high-sensitivity C-reactive protein; hsTnI, high-sensitivity troponin I; MPV, mean platelet volume; PDW, platelet distribution width; PT, prothrombin time; WBC, white blood cell.
