# Supplementary material for: Case Report: Recanalization of Branch Retinal Artery Occlusion Due to Microthrombi Following the First Dose of SARS-CoV-2 mRNA Vaccination
Source: Front Pharmacol. 2022 Mar 24;13:845615. doi: 10.3389/fphar.2022.845615 (PMC8988066; doi:10.3389/fphar.2022.845615)
Supplement: Supplementary file 2 [file Image2.pdf]

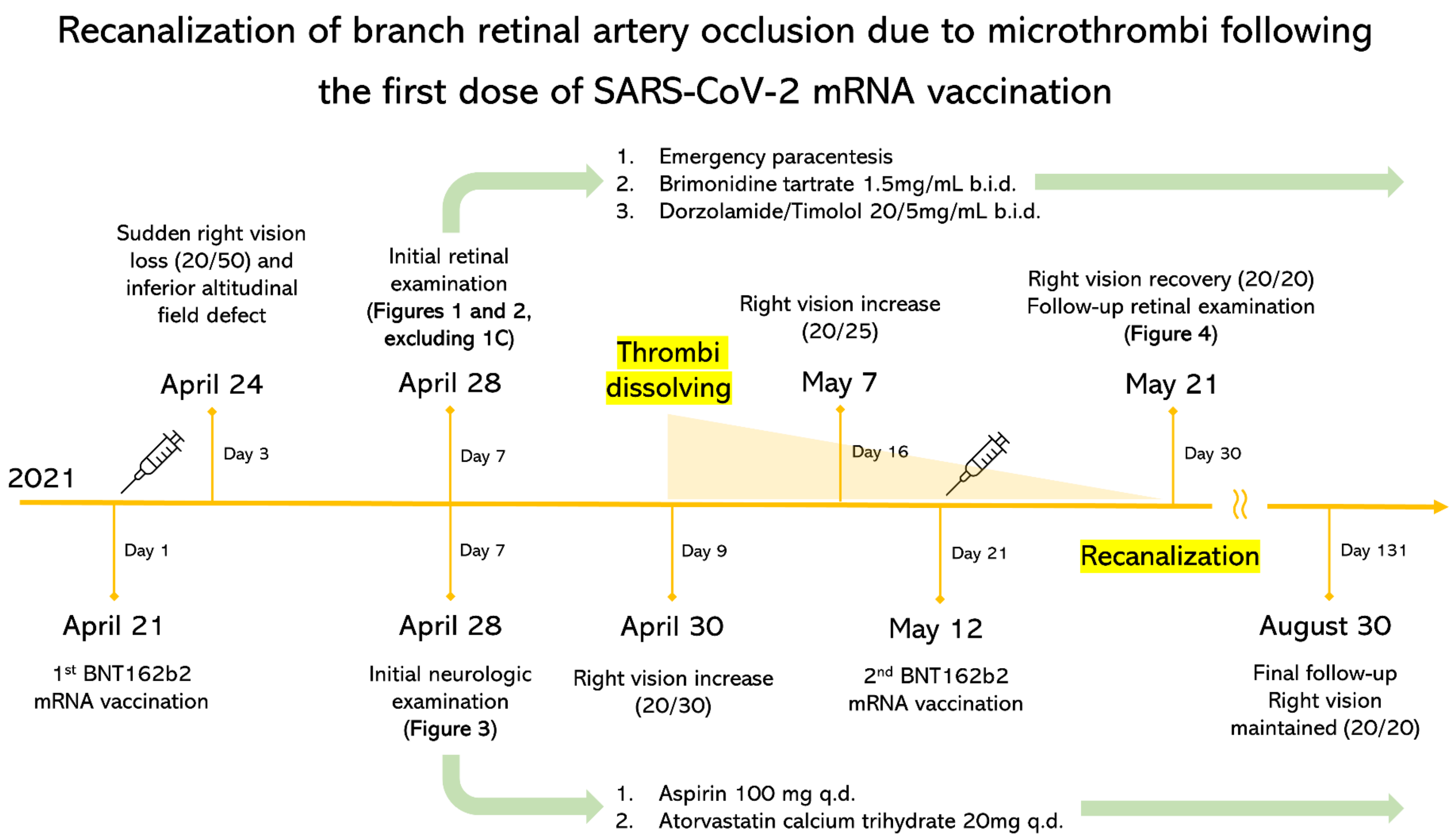

SUPPLEMENTAL FIGURE 2. A timeline of recanalization from branch retinal artery occlusion due to microthrombi following the first SARS-CoV-2 mRNA vaccination Major symptoms and medical events, from the first dose of the BNT162b2 mRNA vaccine on April 21, 2021 to the final follow-up on August 30, 2021, are depicted in the timeline.
