# Supplementary material for: Case Report: Recanalization of Branch Retinal Artery Occlusion Due to Microthrombi Following the First Dose of SARS-CoV-2 mRNA Vaccination
Source: Front Pharmacol. 2022 Mar 24;13:845615. doi: 10.3389/fphar.2022.845615 (PMC8988066; doi:10.3389/fphar.2022.845615)
Supplement: Supplementary file 3 [file Image1.pdf]

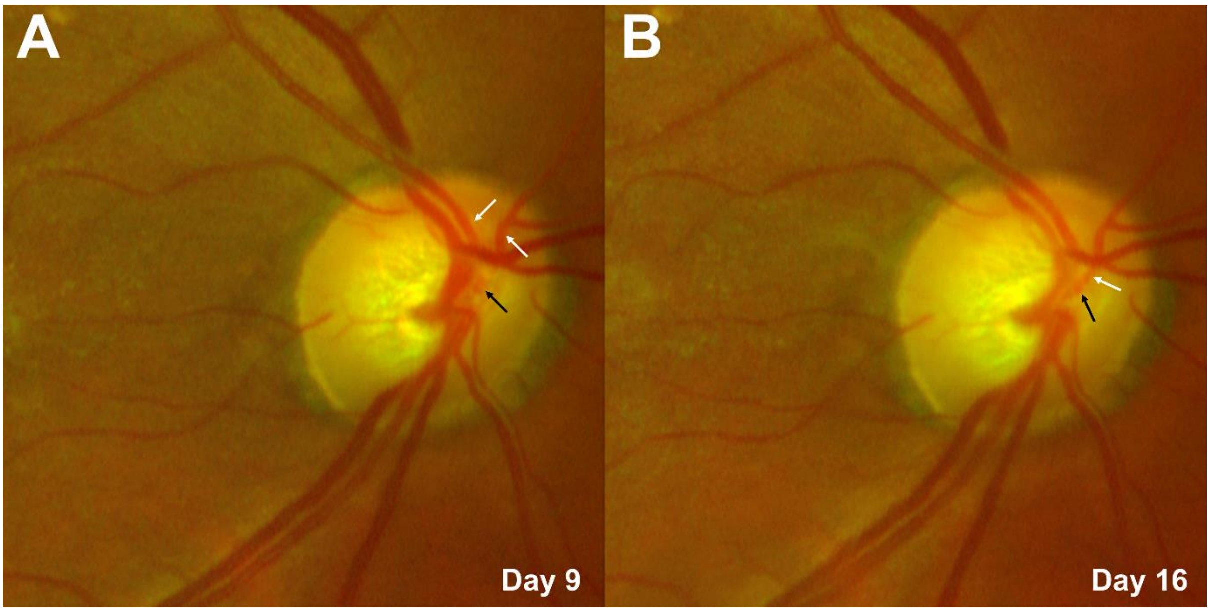

SUPPLEMENTAL FIGURE 1. Continuous dissolution of microthrombi after the initiation of treatment (**A**) On day 7 post-vaccination (2 days after treatment), thrombi in the superotemporal branch dissolved, and arterial flow increased (white arrows). However, remnant thrombi impede arterial blood flow (black arrow). (**B**) As the remaining thrombi progressively dissolved, arterial flow increased on day 16 post-vaccination (11 days after treatment).
